# Predictable Factors of People with Asymmetrical Hearing Loss Wearing a Hearing Aid in the Worse Ear Only

**DOI:** 10.3390/jcm12062251

**Published:** 2023-03-14

**Authors:** Heil Noh, Dong-Hee Lee

**Affiliations:** 1 Department of Otolaryngology-Head and Neck Surgery, St. Vincent’s Hospital, College of Medicine, The Catholic University of Korea, Seoul 06591, Republic of Korea; 2 Department of Otolaryngology-Head and Neck Surgery, Uijeongbu St. Mary’s Hospital, College of Medicine, The Catholic University of Korea, Seoul 11765, Republic of Korea

**Keywords:** hearing aids, hearing devices, outcomes, hearing loss, asymmetry

## Abstract

In patients with bilateral asymmetrical hearing loss (AHL), where only one hearing aid is available, it is difficult to decide which ear to amplify. The aim of this study was to evaluate the outcomes of hearing aid use for AHL patients fitted with a hearing aid in their worse ear only. One-hundred-two adults with asymmetrical-mixed or sensorineural hearing loss were retrospectively included. AHL was classified into three subgroups: unilateral hearing loss (UHL) and AHL type 1 (AHL1) and type 2 (AHL2). The main outcome measures were (1) the time spent wearing a hearing aid, (2) the hearing in a noise test (HINT), (3) the sound localization test and (4) the Korean version of the International Outcome Inventory for Hearing Aids (IOI-HA). The 1 kHz-hearing threshold of the better ear was significantly better in the successful users than in the intermittent users for UHL. Younger age was associated with significantly better outcomes than older for AHL1 and AHL2. Among the etiologies of AHL, sudden hearing loss was associated with significantly better outcomes of hearing aid use for AHL, UHL and AHL1 patients. In this study, the success rate and usage rates were 43.1% and 67.6% in AHL patients wearing a hearing aid in the worse ear. This study identified the hearing threshold of 1 kHz from the better ear, age and etiology of sudden hearing loss as audiometric and non-audiometric factors that affected the outcomes of hearing aid use.

## 1. Introduction

For a long time, there have been many studies to find out the factors which are good enough to consistently differentiate successful from unsuccessful hearing aid candidates [1,2,3,4,5,6,7]. Unfortunately, no factor has been found yet, although certain parameters were statistically significantly related to individual measures of successful hearing aid use [1,2,3,4,5,6,7]. “Two hearing aids work better than one” has been one of the conventional principles in hearing rehabilitation, even though there may be some exceptions [8,9,10,11]. Based on that principle, clinicians often recommend bilateral hearing aids for patients with hearing loss in both ears. Bilateral hearing aids reduce the risk of auditory deprivation of the unaided ear [12,13,14,15,16]. They may also present better discrimination than wearing one hearing aid. Patients who wear two hearing aids do not have to set the amplification unnecessarily high, and they can easily judge where the sound is coming from [17]. For those reasons, clinicians expect higher satisfaction from bilaterally hearing-impaired patients wearing two hearing aids rather than one. However, in cases of asymmetrical hearing loss (AHL), the fitting strategies are less clear, and it is tricky to accomplish satisfactory binaural hearing with either monaural or binaural amplification. Although Briskey’s as well as Arlinger et al.’s recommendations or the 60 dB HL rule (fitting the ear with a four-frequency pure-tone average threshold closer to 60 dB of HL) is straightforward and simple for clinicians [18,19], they tend to underestimate possible advantages of wearing two hearing aids. In addition, Mueller and Hall [20] asserted that the aided threshold values, not the unaided thresholds, should be used to determine whether the patient or the ear is a reasonable candidate for hearing aid(s).

Although it is well-known that two hearing aids are the best for bilateral hearing loss, we often encounter a lot of cases where patients can only afford one hearing aid for bilateral hearing loss for economic reasons. If only one hearing aid is affordable, selecting which ear to amplify in cases with bilateral symmetrical hearing loss is not difficult. However, approaches in selecting which ear to amplify are quite different between unilateral and bilateral hearing aids. We often encounter AHL cases in which binaural symmetry cannot be accomplished with hearing aid(s). In some situations where only one hearing aid is permissible to patients with AHL, the 60 dB HL rule has been a simple, practical guideline but is inadequate when deciding which ear should be fitted with a hearing aid. Because only a few researchers have reported the choice of either ear (better or worse) for monaural amplification in patients with AHL, we have frequently struggled to determine which ear should be fitted for a hearing aid. In many cases of AHL, we have empirically prescribed a hearing aid to the worse ear, based on audiometric data, surveys and patient interviews, and found that our choice was not wrong in most cases. However, we have often encountered cases in which the side fitted with a hearing aid changed from the worse ear to the better ear during the process of counseling and fitting. This discrepancy between the clinician’s recommendation and patient acceptance has been a big problem in real situations.

Our alternative hypothesis was that empirical prescription of a hearing aid to the worse ear in cases with AHL could reduce the interaural difference in the hearing and contribute to better satisfaction for a hearing aid. To prove this hypothesis, we analyzed cases with AHL in which a hearing aid was prescribed to the worse ear. In addition, the secondary objective of this study was to identify any predictable factors that might influence hearing aid outcomes.

## 2. Materials and Methods

### 2.1. Design and Setting

This retrospective observational study recruited Korean adults with AHL who purchased a hearing aid at one secondary-referral, university-based hospital. The inclusion criteria were as follows: (1) 19 years old or above; (2) asymmetrical hearing loss; (3) sensorineural or mixed-type HL; (4) ear anatomy compatible with wearing a conventional hearing aid; and (5) hearing aid fitted to the worse ear. The exclusion criteria were (1) conductive hearing loss; (2) fluctuating hearing loss; (3) observed neurological or psychiatric disorder; (4) inadequate cognitive competence for responding to questionnaires; and (5) self-reported physical or mental health unsuitable for hearing aid fitting and use. Patients with some visual problems, those with tinnitus and those with balance problems were not excluded, but patients belonging to the deaf community and users of sign language were excluded.

The degree and type of hearing loss were classified using the four-frequency (0.5, 1, 2 and 4 kHz) pure-tone average thresholds (PTA4) and the criteria set by the World Health Organization [21]. AHL is a broad term used to describe any degree of interaural asymmetry in hearing thresholds; in the extreme case, the poorer ear presents total deafness, and the contralateral ear presents normal hearing or a mild degree of hearing loss. In this study, we defined AHL as a hearing loss with an interaural difference of 15 dB HL or greater in the PTA4 [22], and we further classified it into four subgroups for a more detailed analysis according to the criteria defined in Table 1, which was modified from previous studies of Van de Heyning and colleagues [22]. Because the single-sided deafness (SSD) subgroup is a poor candidate for a hearing aid, we excluded SSD cases and analyzed cases of three subgroups (unilateral hearing loss (UHL), and asymmetrical hearing loss type 1 (AHL1) and type 2 (AHL2)), which were classified based on the poorer ear PTA4 of 70 dB HL and the better ear PTA4 of 30 dB HL. If the hearing in the worse ear is better than 70 dB HL and the better ear is poorer than 30 dB HL but better than the worse ear by 15 dB HL or more, it is classified as AHL1 (for example, 65 dB HL in the worse ear and 35 dB HL in the better ear). If the hearing in the worse ear is 70 dB HL or more and that of the better ear is poorer than 30 dB HL but better than the worse ear by 15 dB HL, it is classified as AHL2 (for example, 75 dB HL in the worse ear and 55 dB HL in the better ear).

At 1, 2, 3, 6 and 12 months after the purchase, a hearing aid fitting was performed by one author (H.N.) using the National Acoustic Laboratories (NAL) formula. The hearing aid setting was modified and fitted using Noah software (Hearing Instrument Manufacturers’ Software Association (HIMSA)) or the manufacturer’s fitting programs. The fine-tuning procedure was primarily performed to solve the patient’s subjective discomfort. To avoid interference with the better ear, we did not force it to reach the target gain. The real ear-aided response (REAR) was checked with a FONIX FP35 Portable Hearing Aid Analyzer (Frye Electronics. Inc., USA) to verify the proper gain only in case of unusual discomfort. Twelve months post-fitting, the performance of hearing aid use were evaluated and classified into three groups: (1) successful users, (2) intermittent users and (3) failed users. Successful users met all of the following criteria: (1) wearing the hearing aid for more than four hours per day; (2) wearing the hearing aid for twelve months or longer after purchase; (3) showing improved speech discrimination score more than 2% in the hearing in noise test (HINT); (4) showing improved sound localization; and (5) scoring 21 points or more in the International Outcome Inventory for Hearing Aids (IOI-HA) [23]. Patients who stopped using a hearing aid within the first twelve months after initial wearing were classified as failed users. Cases that did not meet the criteria for either successful or failed users were considered intermittent users.

### 2.2. Participants

The clinical files of 102 patients were included in the analysis (37 men and 65 women with an average age of 63 ± 14 years). The demographic data of the participants in this study are presented in Table 2.

This study classified the etiologies of AHL into sudden hearing loss, central nervous system (CNS) problems and other unspecified (or unidentified) etiology. The former two were grouped as retrocochlear/neural causes. Twenty-seven patients (age = 60 ± 14 years; 10 men and 17 women) experienced AHL resulting from sudden hearing loss. Their PTA4s were 65 ± 12 dB HL for the worse ear and 27 ± 14 dB HL for the better ear. Among those 27 patients, 19 were successful users, 3 were intermittent users, and 5 were failed users. Among all 102 patients, three cases resulted from a CNS problem (age = 72 ± 8 years; one man and two women). Their PTA4s were 68 ± 10 dB HL for the worse ear and 31 ± 10 dB HL for the better ear. One patient was a successful user, and two were intermittent users.

This study defined the success rate of a hearing aid as the percentage of successful users who bought a hearing aid and the usage rate as the percentage of successful and intermittent users to patients who bought a hearing aid.

### 2.3. Interviews and Counseling for Amplification

Patient selection, interviews for amplification, choice of the aided ear and hearing aid selection and fitting were performed by one author (H.N.). The interview was simply structured, and two questions were developed for AHL. In the beginning, the routine hearing aid counseling styles were similar, putting hearing aids in both ears or to the worse ear. Whoever thinks that their better ear has a normal hearing range and decides to have one hearing aid for the worse ear only, we asked them if the amplified worse ear hears similar loudness compared to the better ear. After the final fitting reached the target gain, the second question was if the client perceived the binaural symmetry. The first question focused on loudness, and the second was focused on space perception or stereo. Most of the clients could express the loudness differences or similarities. Some had difficulties expressing symmetry at first. However, during the follow-up periods, most patients perceived and expressed that they had gained some symmetry.

### 2.4. Outcome Measures

After 12 months post-fitting, four main outcomes were administered: (1) the time wearing a hearing aid, which was investigated using IOI-HA as well as data-logging, (2) the HINT at a fixed intensity at SNR 0 dB, (3) sound localization test at a fixed level of 50 dB and (4) the Korean version of the IOI-HA.

The HINT and sound localization were performed in unaided and aided conditions. The HINT was performed in a double-walled, sound-treated booth, and 50 recorded Korean monosyllables were presented using presentation levels from 40 dB in 5 dB steps at a 1 m distance in the sound field while speech spectrum noise was presented with a fixed intensity at SNR 0 dB. The aided–unaided difference in speech discrimination score of more than 2% was a criterion of meaningful difference. The sound localization test was performed in a double-walled, sound-treated booth, and the spondee was presented at a fixed level of 50 dB at 1 m in azimuth of the front, right and left sides in the sound field while the presented azimuth was randomly selected, and the speech was presented during 5 s with a silent interval of 5 s. The examinee answered the direction of a sound source. If the examinee responded to all three directions correctly, we decided that he or she would receive a sound localization. This study defined improved sound localization in which a test result was positive in aided condition regardless of an unaided result.

### 2.5. Statistical Analysis

The probability value of *p* < 0.05 was considered to be statistically significant. All statistical analyses were performed using IBM^®^-SPSS^®^ statistics (version 21, IBM Corporation, New York, NY, USA). The data are expressed as the mean ± standard deviation. Differences in numerical values (age, PTA4) were examined using the Kruskal–Wallis test for the three outcomes of hearing-aid use and the Mann–Whitney U test for comparing two outcomes (successful and intermittent users versus failed users) in multiple independent samples separated by age, the threshold at each frequency and PTA4. Differences in ordinary values (sex, aided side, type of hearing loss and etiology) among the three outcomes for hearing aid use were tested using the chi-squared test in subgroups separated by sex, aided side, type of hearing loss, the subtype of AHL and etiology of hearing loss. A discriminant analysis was performed as a multivariate test of differences among the three outcomes (successful versus intermittent versus failed users) and between two outcomes (successful and intermittent users versus failed users).

## 3. Results

Forty-four patients were successful users, twenty-five patients were intermittent users and thirty-three patients were failed users. The success rate of a hearing aid worn in the worse ear in cases with AHL was 43.1% (=44/102) in this study. The usage rate was 67.6% (=69/102).

### 3.1. Analysis of Outcome Differences among All Cases (n = 102)(Figure S1)

**Figure 1:**
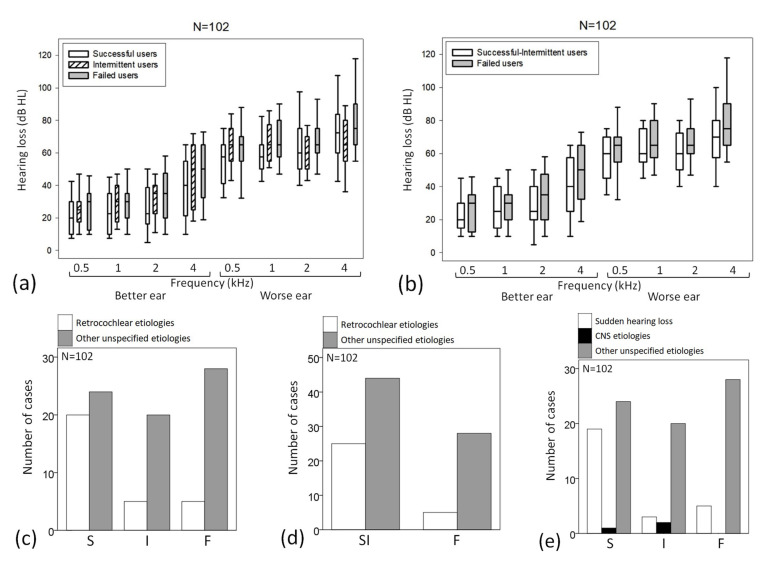
(**a**–**e**) In all cases (n = 102), box plots of differences in the hearing thresholds according to (**a**) three and (**b**) two outcomes. Comparison by the etiology of hearing loss according to (**c**,**e**) three and (**d**) two outcomes.

Among the parameters (age, threshold at each frequency, PTA4, sex, lesion site, type of hearing loss, the subgroup of AHL and etiology of hearing loss), only age and etiology significantly differed among the three or two outcomes of hearing aid use. Successful users were younger than intermittent users (*p* = 0.041; 59 ± 13 years old for successful users; 67 ± 12 years old for intermittent users; and 65 ± 15 years old for failed users). No other frequencies significantly differed with three outcomes (Figure 1a) or two outcomes (Figure 1b). Compared with the other unspecified etiologies, the retrocochlear/neural etiology of hearing loss was found more often for successful users than for the other two users (*p* = 0.008; Figure 1c) and also found successful-intermittent users more often than for the failed users (*p* = 0.037; Figure 1d). Compared with CNS or other unspecified causes, sudden hearing loss was found more often for successful users than for the other two user groups (*p* = 0.006) (Figure 1e).

For three outcomes (successful users versus intermittent users versus failed users), the discriminant function was D = −2.886 + (1.182 × etiology; sudden hearing loss versus CNS versus other unspecified causes; and discriminating power = 47.1%). For two outcomes (successful-intermittent users versus failed users), the discriminant function was D = −3.797 + (2.226 × etiology; retrocochlear/neural versus other unspecified causes; and discriminating power = 52.0%).

### 3.2. Analysis of Outcome Differences in the UHL Subgroup (n = 48)(Figure S2)

**Figure 2:**
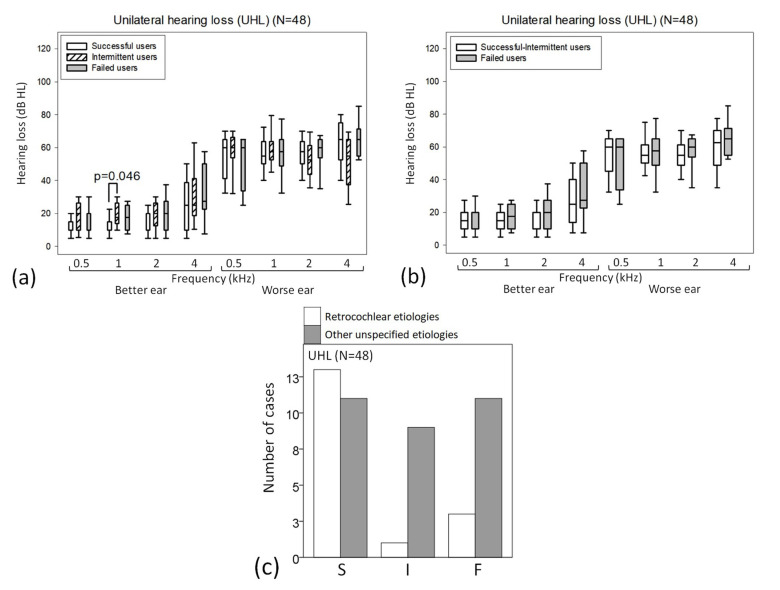
(**a**–**c**) In the UHL subgroup (n = 48), box plots of differences in the hearing thresholds according to (**a**) three and (**b**) two outcomes. Comparison by the etiology of hearing loss according to (**c**) three outcomes.

The hearing threshold at 1 Hz for the better ear was lower for successful users than for intermittent users (*p* = 0.046; 13 ± 6 dB HL for successful users; 19 ± 7 dB HL for intermittent users; and 17 ± 8 dB HL for failed users) (Figure 2a,b). Compared with the other unspecified etiologies, the retrocochlear/neural etiology of hearing loss was found more often for successful users than for the other two outcomes (*p* = 0.021) (Figure 2c).

For three outcomes (successful user versus intermittent user versus failed users), the discriminant function was D = −3.637 + (2.210 × etiology; retrocochlear/neural versus other unspecified causes; and discriminating power = 45.8%).

### 3.3. Analysis of Outcome Differences in the AHL1 Subgroup (n = 26) (Figure S3)

**Figure 3:**
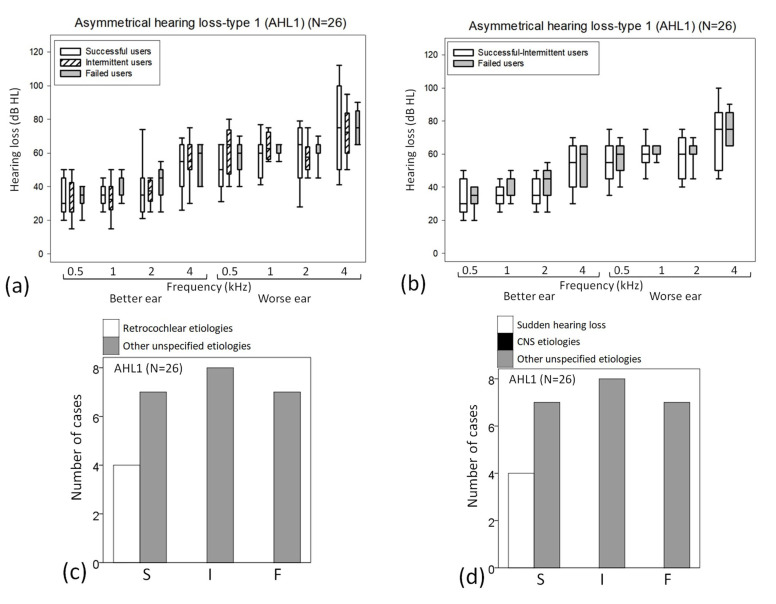
(**a**–**d**) In the AHL1 subgroup (n = 26), box plots of differences in the hearing thresholds according to (**a**) three and (**b**) two outcomes. Comparison by the etiology of hearing loss according to (**c**,**d**) three outcomes.

In the AHL1 subgroups, no specific frequency significantly differed with three outcomes (Figure 3a) or two outcomes (Figure 3b). Compared with the other unspecified etiologies, the retrocochlear/neural etiology of hearing loss was found more often for successful users than for intermittent or failed users (*p* = 0.040) (Figure 3c). Compared with CNS or other unspecified causes, sudden hearing loss was found more often for successful users than for the other two outcome groups (*p* = 0.040) (Figure 3d). No significant discriminant function was found with three outcomes or two outcomes.

### 3.4. Analysis of Outcome Differences in the AHL2 Subgroup (n = 28) (Figure S4)

**Figure 4:**
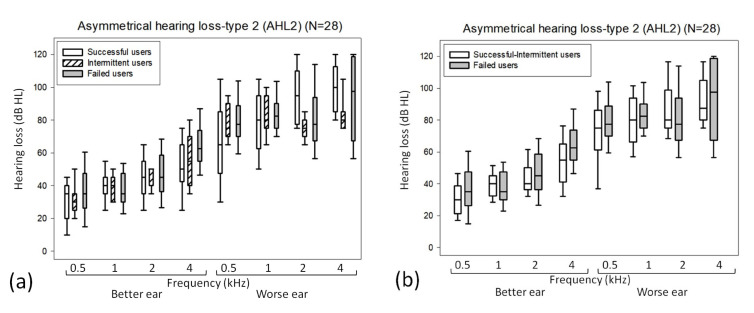
(**a**,**b**) Box plots of differences in the hearing thresholds according to (**a**) three and (**b**) two outcomes for the AHL2 subgroup (n = 28).

Successful users were younger than intermittent and failed users (*p* = 0.007; successful users, 63 ± 11 years old; intermittent users, 76 ± 4 years old; and failed users, 73 ± 8 years old). For the AHL2 subgroup, no specific frequency significantly differed with three outcomes (Figure 4a) or two outcomes (Figure 4b). For three outcomes (successful users versus intermittent users versus failed users), the discriminant function was D = −8.372 + (0.119 × age) (discriminating power = 53.6%).

## 4. Discussion

In 2019, an estimated 1.57 billion people had hearing loss worldwide, which accounted for one in five global people. Of these, 430.4 million people did not use a hearing aid, and 403.3 million people had moderate hearing loss or deafness in the better-hearing ear [24]. These people are most likely to benefit from clinical attention and interventions, such as hearing aids. However, the usage rate of a hearing aid was very low in the past. The 1999–2006 cycles of the National Health and Nutritional Examination Surveys (NHANES) in the United States found that the prevalence of hearing aid use in people with a hearing loss of 25 dB HL or more was 4.3% in 50–59 year-olds as well as 22.1% in 80 years and older people [25]. The 2005–2006 cycle of the NHANES in the Unites States surveyed that hearing aids were used in 40.0% of adults with moderate hearing loss but in only 3.4% of those with mild hearing loss (overall prevalence of hearing aid use = 19.1%) [26]. These two surveys defined hearing aid use as wearing at least once a day (1999–2004) or for at least 5 h per week, using an interviewer-administered questionnaire. The prevalence of hearing aid usage decreased in certain group of hearing-impaired people; for example, for people with UHL, it was 2.0% (1.4% of those with mild UHL and 4.2% of those with moderate UHL) in the studies from the 2005 to 2006, 2009 to 2010 and 2011 to 2012 cycles of the NHANES [27]. Factors suggested as reasons for low use of a hearing aid include the denial of hearing loss, cost for buying and maintenance, discomfort resulting from ear plugging, frequent infection of the external ear, aggravated draining in the case of chronic otitis media, a perceived lack of benefit and a perceived stigma associated with the hearing aids use. Many studies have dealt with this topic [4,5,28,29,30,31,32,33,34] and Lauer et al. [35] proposed the Assistive Technology Interruption Model (ATIM) to understand the discontinuance of assistive technology devices better. Lauer and Smith’s ATIM suggested three factors that can contribute to an interruption in assistive technology use: (1) device-related factors, (2) user-related factors and (3) environmental factors. Recently, the discontinuance of assistive technology, including a hearing aid, has been rising as one of many social concerns, and the WHO coordinates with the Global Cooperation on Assistive Technology (GATE). However, people with hearing loss are changing and motivated. The MarkeTrak 2022 survey, the largest-scale survey that the Hearing Industries Association has regularly conducted in the United States, stated that overall satisfaction with hearing instruments was 83% and ownership of binaural hearing aids was 70% for those fitted in person. It shows that 64% of respondents reported “regular” quality-of-life benefits with hearing aids, but 9% and 2% stated their quality of life improved rarely and never, respectively, even though they were current owners [36].

This study showed that the success rate and usage rates were 43.1% and 67.6%, respectively, in AHL patients wearing a hearing aid in the worse ear. This was much higher than previous reports in South Korea. Moon et al. [37] analyzed a large number of data from the 2010 to 2012 Korea National Health and Nutrition Examination Surveys and reported that 12.6% of patients with bilateral moderate-to-profound sensorineural hearing loss (PTA4 > 40 dB in both ears) used hearing aids regularly in their daily life. Recently, Lee et al. [16] analyzed a large number of data from the 2009 to 2012 Korea National Health and Nutrition Examination Surveys and reported that only 0.86% of patients with unilateral hearing loss (PTA4 ≥ 41 dB in the worse hearing ear and < 41 dB in the other ear) used a hearing aid regularly in their daily life. Unfortunately, this result is not enough to support that a hearing aid fitted to the worse ear in cases with AHL can give better satisfaction for a hearing aid than the general population wearing hearing aid(s) because three studies have different study designs as well as participants. Especially, Lee et al.’s study [16] included SSD cases (21.2% of overall participants), which were excluded from our study. Our study showed that the success and usage rates were 50.0% and 70.8% in UHL, 42.3% and 73.1% in AHL1, and 32.1% and 57.1% in AHL2, which were much higher than the usage rates published in the previous reports. Our hypothesis was that a hearing aid worn in the worse ear could attribute better satisfaction in AHL patients, and this study demonstrated that this hypothesis was true. A recent study reported that the mixed state of asynchrony and synchrony was reversible for asymmetric hearing loss with an interaural threshold from 15 dB to 40 dB, and amplification of the poorer ear improved the performance of hearing in noise and normalized interhemispheric temporal organization [38,39].

Because many hearing aid fitting formulae have been developed on the assumption of similar hearing loss between the two ears, otologists and audiologists always encounter the following questions while counseling patients with AHL: (1) Should we recommend unilateral or bilateral hearing aids to them? (2) If only one hearing aid is preferable or feasible, should the better or worse ear be fitted? In South Korea, many patients with symmetrical or asymmetrical hearing loss buy only one hearing aid, and the second question is a serious dilemma in real clinical situations. For monaural amplification in AHL, two self-questions are useful in selecting the ear to be aided. (1) If the better ear is aided, can aided hearing improve speech detection as well as understanding for the AHL patient? (2) If the worse ear is aided, can the patient understand better in quiet as well as in noisy environments and obtain the advantages of binaural hearing? Actually, these two questions address the same issue: which ear sends higher-quality signals to the brain when aided? In answering that question, the 60 dB HL rule offers a simple, practical principle for fitting a monaural hearing aid to an AHL patient. Briskey [18] stated the criteria for successful binaural fitting, and Arlinger et al. [19] suggested similar criteria, but these tend to underestimate the possible advantages of wearing two hearing aids [40]. Therefore, the secondary objective of this study was to evaluate any parameter that might predict the successful use of a hearing aid monaurally fitted in the worse ear.

Only the hearing threshold at 1 kHz in the better ear in the UHL group was significantly lower (better) for those who continued hearing aid use. Why is the hearing threshold on the better ear (not wearing a hearing aid) significantly lower? This is not a common-sense result, but it is interesting. The possible explanation comes from the main role of a patient’s better ear in hearing and listening. In many patients with AHL who are monaurally fitted in the worse ear, aided hearing in the worse ear may remain supplementary, especially in situations in which the sound of interest is soft and comes from the side of the aided ear. While speaking in a group conversation or where there are multiple talkers or noises, the better ear can still play the main and crucial role [40]. Our finding was compatible with Boymans et al.’s study [18] but against Lee et al.’s study [16]. Boymans et al. [18] found that unilateral hearing aid was more used than bilateral hearing aids in cases with mild hearing losses in the better ear and that bilateral hearing aids were more used in cases with moderate and severe hearing losses in the better ear. However, Lee et al. [16] stated that patients with more severe hearing loss in both the better ear and the worse ear seemed to adopt a hearing aid. This disagreement might be a result of the difference in the study design as well as the participants’ characteristics.

A second interesting finding was that age influenced hearing-aid use for all cases and patients classified as AHL2 cases. Three subgroups of UHL, AHL1 and AHL2 are quite distinct from each other. UHL seems to be suitable for monaural amplification, but AHL1 is likely to be a candidate for binaural amplification. AHL2 seems to be a dilemma for the selection of which ear to amplify monaurally; if the worse ear is amplified, binaural symmetry can be accomplished, but both ears’ hearing is more likely to be handicapped. If the better ear is amplified, the patient may only receive one serviceable hearing ear. The heterogeneity of both hearing thresholds in cases with AHL2 could explain why younger patients with AHL2 showed better hearing aid use outcomes than older patients. The influence of age may be explained by younger patients’ lifestyles and listening environments, but this study did not analyze them. Because younger patients are likely to have better binaural central processing and better plasticity of the CANS [41], they may be more likely to overcome some of the discomforts that result from monaural amplification of the worse ear in AHL2.

A major limitation of this study was its retrospective design. Because this study included only AHL cases wearing a hearing aid in the worse ear, we cannot present any evidence to determine which ear, better or worse, should be fitted with a unilateral hearing aid in AHL. The second limitation is the subgrouping of AHL. We defined them based on the degree of hearing loss. Many authors have stated that the shape of hearing loss (e.g., up-slope, down-slope, ski-slope and flat) should be considered because the amplification range of hearing aids can be limited according to the frequency range. This study analyzed the parameters among three subgroups but did not consider the differences in demographics (e.g., cognitive function, physical issues, social status and communicative lifestyle). For example, some patients only needed to wear their hearing aid for less than 4 h per day while at work or in a classroom but managed well otherwise, therefore making them not necessarily ‘intermittent’ users. Other issues to consider might include which environment the patient spent most of their day. If they worked in a very noisy factory, it would make sense that they remove the hearing aid while working to avoid amplification of noise. This was why this study analyzed the parameters between two outcomes (successful-intermittent users and failed users). Considering this limitation, we also restricted ‘failed users’ to cases in which a patient returns his or her hearing aid or gives up wearing it. In this study, the etiologies of AHL were classified into three groups (sudden hearing loss, CNS problem and other unspecified). For an individual, the exact specific etiology of hearing loss cannot be easily evaluated because many causes have been mixed, including aging, noise, head trauma, ototoxic drug and so forth. So, we had to focus on specific etiology, which could be sorted quite exactly based on the history taken and the past imaging studies. When evaluating the outcome of a hearing aid, the time of use with the hearing aids is an important factor, as is the frequency of a hearing aid use or the contexts of use. Although the frequency of hearing aid use is also important, this parameter may be too difficult to be used as a comparison variable because of too-large individual variation and too much recall bias. The contexts of hearing aid use are very important, but we did not analyze them because of too much recall bias, and it was too difficult to classify and standardize them as a comparison variable. This study included IOI but not specific instruments to evaluate the outcomes of satisfaction with hearing aid use, such as Expected Consequences of Hearing Aid Ownership (ECHO), Satisfaction with Amplification in Daily Life (SADL), Quebec User Evaluation of Satisfaction with assistive Technology (QUEST) or Psychosocial Impact of Assistive Devices Scale (PIADS). We hope that future study considers these two limitations of this study.

There are too many factors influencing hearing/listening as well as hearing aid performance, such as, for example, audiometric factors (e.g., type, degree and configuration of hearing loss), personal factors (e.g., motivation, expectation, stigma and previous experience with devices), demographic factors (e.g., age, sex, job and listening environment) and external factors (e.g., cost and counseling) [4,5,36]. A lot of previous studies included personal and demographic factors, but few studies evaluated specific audiometric data. Because this study included only audiometric as well as medical factors, it had another limitation but evaluated the data that had not been analyzed in the previous literature, which was already studied and conducted by hearing aid dispensers or manufacturers.

Unfortunately, we failed to find any specific hard-and-fast fitting rules for AHL in this study. It means that the only way to judge whether patients with AHL can successfully use a hearing aid in their worse ear is to fit a hearing aid, give them time to adapt, and then make appropriate adjustments. We hope that our results may provide further evidence supporting the efficacy of unilateral hearing aid fitting to the worse ear in patients with AHL.

## 5. Conclusions

In this study, the success and usage rates were 43.1% and 67.6% in AHL patients wearing a hearing aid in the worse ear, which was much higher than those of previous data in South Korea.

This study found audiometric and non-audiometric factors as predictable factors affecting the outcomes of hearing aid use. Only one audiometric factor in AHL affected the outcomes of hearing aid use: the hearing threshold of 1 kHz of the better ear had statistically significant effects on the success of hearing aid use in UHL. As the non-audiometric factor, age was statistically significant in AHL2. The etiology of sudden hearing loss was another non-audiometric factor that had statistically significant effects on the outcome for patients with AHL, especially in UHL.

## Figures and Tables

**Table 1:** Audiological criteria for the classification of AHL into three subgroups based on the PTA4. This study included only cases with an interaural difference of 15 dB HL or greater in the four-frequency (0.5, 1, 2 and 4 kHz) pure-tone average thresholds.

		Better Ear	
		≤30 dB HL	>30 dB HL
Worse ear	<70 dB HL	Unilateral hearing loss (UHL)	Asymmetrical hearing loss type 1 (AHL1)
	≥70 dB HL	Single-sided deafness (SSD)	Asymmetrical hearing loss type 2 (AHL2)

**Table 2:** Demographic data for the three AHL subgroups of 102 cases (expressed as the mean ± SD).

	Unilateral Hearing Loss (UHL)	Asymmetrical Hearing Loss Type 1 (AHL1)	Asymmetrical Hearing Loss Type 2 (AHL2)
Number of patients	48	26	28
Age (years)	56 ± 15	68 ± 9	70 ± 10
Sex (men:women)	15:33	10:16	12:16
PTA4 of aided (worse) ear (dB HL)	57 ± 7	62 ± 6	83 ± 12
PTA4 of the unaided (better) ear (dB HL)	19 ± 8	40 ± 6	44 ± 9
Interaural threshold difference of PTA_4_ (dB HL)	38 ± 11	22 ± 6	39 ± 15
WRS of aided (worse) ear (%)	67 ± 27	67 ± 20	33 ± 15
Type of hearing loss (sensorineural:mixed)	39:9	17:9	17:11
Hearing-aid type (CIC:ITC:RIC:BTE)	37:0:11:0	22:1:3:0	22:0:2:4
Outcome of hearing aid use (successful:intermittent:failed users)	24:10:14	11:8:7	9:7:12

PTA4: the four-frequency (0.5, 1, 2 and 4 kHz) pure-tone average thresholds on pure-tone audiogram; and WRS: word recognition score.

## Data Availability

Not applicable.

## Supplementary Materials

The following supporting information can be downloaded at: https://www.mdpi.com/article/10.3390/jcm12062251/s1; Figure S1. Key data of box plots of differences in the hearing thresholds and of comparison by the etiology of hearing loss in all cases (n = 102); Figure S2. Key data of box plots of differences in the hearing thresholds and comparison by the etiology of hearing loss in the unilateral hearing loss (UHL) subgroup (n = 48); Figure S3. Key data of box plots of differences in the hearing thresholds according to and comparison by the etiology of hearing loss according to the asymmetrical hearing loss type 1 (AHL1) subgroup (n = 26); Figure S4. Key data of box plots of differences in the hearing thresholds in the asymmetrical hearing loss type 2 (AHL2) subgroup (n = 28).

## Abbreviations

AHL	Asymmetrical hearing loss
AHL1	Asymmetrical hearing loss type 1
AHL2	Asymmetrical hearing loss type 2
UHL	Unilateral hearing loss
HINT	Hearing in noise test
IOI-HA	International Outcome Inventory for Hearing Aids.
