# Supplementary material for: Predictable Factors of People with Asymmetrical Hearing Loss Wearing a Hearing Aid in the Worse Ear Only
Source: J Clin Med. 2023 Mar 14;12(6):2251. doi: 10.3390/jcm12062251 (PMC10059651; doi:10.3390/jcm12062251)
Supplement: Supplementary file 1 [file jcm-12-02251-s001.zip › jcm-2255086-supplementary.pdf]

Supplementary Materials:

Figure S1. Key data of box-plots of differences in the hearing thresholds and of comparison by the etiology of hearing loss in all cases (n=102).

(a) Box-plots of differences in the hearing thresholds according to three outcomes (successful users, intermittent users and failed users).

		N	Mean	Standard deviation	Standard error	95% Confidence interval for mean		Minimum	Maximum
						Lower bound	Upper bound		
Better ear; 0.5kHz	Successful users	44	21.4773	12.83072	1.93430	17.5764	25.3782	.00	50.00
	Intermittent users	25	26.0000	11.36515	2.27303	21.3087	30.6913	5.00	50.00
	Failed users	33	26.0606	14.45670	2.51659	20.9345	31.1867	5.00	65.00
	Total	102	24.0686	13.12036	1.29911	21.4915	26.6457	.00	65.00
Better ear; 1kHz	Successful users	44	23.7500	13.98192	2.10785	19.4991	28.0009	5.00	55.00
	Intermittent users	25	29.4000	12.27464	2.45493	24.3333	34.4667	10.00	50.00
	Failed users	33	28.7879	13.11192	2.28249	24.1386	33.4372	5.00	55.00
	Total	102	26.7647	13.43571	1.33033	24.1257	29.4037	5.00	55.00
Better ear; 2kHz	Successful users	44	27.3864	17.06651	2.57287	22.1977	32.5751	.00	80.00
	Intermittent users	25	31.4000	12.45994	2.49199	26.2568	36.5432	5.00	50.00
	Failed users	33	34.6970	17.45259	3.03811	28.5086	40.8854	5.00	70.00
	Total	102	30.7353	16.35614	1.61950	27.5226	33.9479	.00	80.00
Better ear; 4kHz	Successful users	44	37.7273	20.97769	3.16251	31.3495	44.1051	.00	80.00
	Intermittent users	25	45.6000	19.96455	3.99291	37.3590	53.8410	10.00	80.00
	Failed users	33	48.4848	20.51999	3.57207	41.2088	55.7609	5.00	90.00
	Total	102	43.1373	20.95400	2.07476	39.0215	47.2530	.00	90.00
Better ear; Mean	Successful users	44	27.5852	13.74973	2.07285	23.4049	31.7655	2.50	52.50
	Intermittent users	25	33.1000	11.69891	2.33978	28.2709	37.9291	10.00	52.50
	Failed users	33	34.5076	14.55371	2.53348	29.3471	39.6681	7.50	65.00
	Total	102	31.1765	13.79417	1.36583	28.4670	33.8859	2.50	65.00
Worse ear; 0.5kHz	Successful users	44	55.5682	16.53804	2.49320	50.5402	60.5962	30.00	105.00
	Intermittent users	25	64.6000	14.64297	2.92859	58.5557	70.6443	30.00	95.00
	Failed users	33	63.0303	18.66394	3.24898	56.4124	69.6482	25.00	110.00
	Total	102	60.1961	17.16146	1.69924	56.8252	63.5669	25.00	110.00
Worse ear; 1kHz	Successful users	44	60.6818	15.38615	2.31955	56.0040	65.3596	35.00	105.00
	Intermittent users	25	67.0000	14.06829	2.81366	61.1929	72.8071	45.00	100.00
	Failed users	33	67.2727	17.18879	2.99218	61.1778	73.3676	20.00	105.00
	Total	102	64.3627	15.86876	1.57124	61.2458	67.4797	20.00	105.00
Worse ear; 2kHz	Successful users	44	64.0909	20.43915	3.08132	57.8768	70.3050	25.00	120.00
	Intermittent users	25	60.2000	13.10852	2.62170	54.7891	65.6109	35.00	85.00
	Failed users	33	66.9697	17.22731	2.99889	60.8612	73.0782	30.00	120.00
	Total	102	64.0686	17.86543	1.76894	60.5595	67.5777	25.00	120.00
Worse ear; 4kHz	Successful users	44	72.2727	22.52788	3.39621	65.4236	79.1218	30.00	120.00
	Intermittent users	25	67.2000	19.20720	3.84144	59.2717	75.1283	25.00	105.00
	Failed users	33	77.2727	20.35202	3.54283	70.0562	84.4892	50.00	120.00
	Total	102	72.6471	21.19810	2.09892	68.4834	76.8108	25.00	120.00
Worse ear; Mean	Successful users	44	63.1534	14.04025	2.11665	58.8848	67.4220	41.25	110.00
	Intermittent users	25	64.7500	12.08154	2.41631	59.7630	69.7370	46.25	96.25
	Failed users	33	68.6364	15.12759	2.63338	63.2724	74.0004	46.25	113.75
	Total	102	65.3186	14.02977	1.38915	62.5629	68.0743	41.25	113.75

(b) Box-plots of differences in the hearing thresholds according to two outcomes (successful-intermittent users and failed users).

		N	Mean	Standard deviation	Standard error	95% Confidence interval for mean		Minimum	Maximum
						Lower bound	Upper bound		
Better ear; 0.5kHz	Successful-Intermittent users	69	23.1159	12.42925	1.49631	20.1301	26.1018	.00	50.00
	Failed users	33	26.0606	14.45670	2.51659	20.9345	31.1867	5.00	65.00
	Total	102	24.0686	13.12036	1.29911	21.4915	26.6457	.00	65.00
Better ear; 1kHz	Successful-Intermittent users	69	25.7971	13.57504	1.63424	22.5360	29.0582	5.00	55.00
	Failed users	33	28.7879	13.11192	2.28249	24.1386	33.4372	5.00	55.00
	Total	102	26.7647	13.43571	1.33033	24.1257	29.4037	5.00	55.00
Better ear; 2kHz	Successful-Intermittent users	69	28.8406	15.58055	1.87568	25.0977	32.5834	.00	80.00
	Failed users	33	34.6970	17.45259	3.03811	28.5086	40.8854	5.00	70.00
	Total	102	30.7353	16.35614	1.61950	27.5226	33.9479	.00	80.00
Better ear; 4kHz	Successful-Intermittent users	69	40.5797	20.82024	2.50646	35.5781	45.5813	.00	80.00
	Failed users	33	48.4848	20.51999	3.57207	41.2088	55.7609	5.00	90.00
	Total	102	43.1373	20.95400	2.07476	39.0215	47.2530	.00	90.00
Better ear; Mean	Successful-Intermittent users	69	29.5833	13.22818	1.59249	26.4056	32.7611	2.50	52.50
	Failed users	33	34.5076	14.55371	2.53348	29.3471	39.6681	7.50	65.00
	Total	102	31.1765	13.79417	1.36583	28.4670	33.8859	2.50	65.00
Worse ear; 0.5kHz	Successful-Intermittent users	69	58.8406	16.36318	1.96990	54.9097	62.7714	30.00	105.00
	Failed users	33	63.0303	18.66394	3.24898	56.4124	69.6482	25.00	110.00
	Total	102	60.1961	17.16146	1.69924	56.8252	63.5669	25.00	110.00
Worse ear; 1kHz	Successful-Intermittent users	69	62.9710	15.12980	1.82141	59.3364	66.6056	35.00	105.00
	Failed users	33	67.2727	17.18879	2.99218	61.1778	73.3676	20.00	105.00
	Total	102	64.3627	15.86876	1.57124	61.2458	67.4797	20.00	105.00
Worse ear; 2kHz	Successful-Intermittent users	69	62.6812	18.12091	2.18150	58.3280	67.0343	25.00	120.00
	Failed users	33	66.9697	17.22731	2.99889	60.8612	73.0782	30.00	120.00
	Total	102	64.0686	17.86543	1.76894	60.5595	67.5777	25.00	120.00
Worse ear; 4kHz	Successful-Intermittent users	69	70.4348	21.38133	2.57401	65.2984	75.5711	25.00	120.00
	Failed users	33	77.2727	20.35202	3.54283	70.0562	84.4892	50.00	120.00
	Total	102	72.6471	21.19810	2.09892	68.4834	76.8108	25.00	120.00
Worse ear; Mean	Successful-Intermittent users	69	63.7319	13.29545	1.60058	60.5380	66.9258	41.25	110.00
	Failed users	33	68.6364	15.12759	2.63338	63.2724	74.0004	46.25	113.75
	Total	102	65.3186	14.02977	1.38915	62.5629	68.0743	41.25	113.75

(c) Comparison by the etiology of hearing loss according to three outcomes (successful users, intermittent users and failed users).

		Etiology		
		Retrocochlear/neural causes	Other unspecified causes	Total
Outcomes	Successful users	20	24	44
	Intermittent users	5	20	25
	Failed users	5	28	33
Total		30	72	102

(d) Comparison by the etiology of hearing loss according to two outcomes (successful-intermittent users and failed users).

		Etiology		
		Retrocochlear/neural causes	Other unspecified causes	Total
Outcomes	Successful-Intermittent users	25	44	69
	Failed users	5	28	33
Total		30	72	102

(e) Comparison by the etiology of hearing loss according to three outcomes (successful users, intermittent users and failed users).

		Etiology			
		Sudden hearing loss	CNS causes	Other unspecified causes	Total
Outcomes	Successful users	19	1	24	44
	Intermittent users	3	2	20	25
	Failed users	5	0	28	33
Total		27	3	72	102

Figure S2. Key data of box-plots of differences in the hearing thresholds and comparison by the etiology of hearing loss in unilateral hearing loss (UHL) subgroup (n = 48).

(a) Box-plots of differences in the hearing thresholds according to three outcomes (successful users, intermittent users and failed users).

		N	Mean	Standard deviation	Standard error	95% Confidence interval for mean		Minimum	Maximum
						Lower bound	Upper bound		
Better ear; 0.5kHz	Successful users	24	12.9167	6.24094	1.27393	10.2814	15.5520	.00	30.00
	Intermittent users	10	19.0000	9.06765	2.86744	12.5134	25.4866	5.00	30.00
	Failed users	14	14.6429	8.19575	2.19041	9.9108	19.3749	5.00	30.00
	Total	48	14.6875	7.67742	1.10814	12.4582	16.9168	.00	30.00
Better ear; 1kHz	Successful users	24	12.7083	5.70644	1.16482	10.2987	15.1179	5.00	25.00
	Intermittent users	10	19.0000	7.37865	2.33333	13.7216	24.2784	10.00	30.00
	Failed users	14	17.1429	7.52286	2.01057	12.7993	21.4864	5.00	30.00
	Total	48	15.3125	7.02626	1.01415	13.2723	17.3527	5.00	30.00
Better ear; 2kHz	Successful users	24	15.8333	7.61387	1.55417	12.6183	19.0484	.00	30.00
	Intermittent users	10	19.5000	8.95979	2.83333	13.0906	25.9094	5.00	30.00
	Failed users	14	20.0000	11.09400	2.96500	13.5945	26.4055	5.00	40.00
	Total	48	17.8125	9.04517	1.30556	15.1861	20.4389	.00	40.00
Better ear; 4kHz	Successful users	24	26.4583	18.50259	3.77683	18.6454	34.2713	.00	80.00
	Intermittent users	10	29.5000	16.23611	5.13431	17.8854	41.1146	10.00	65.00
	Failed users	14	32.1429	17.17716	4.59079	22.2251	42.0607	5.00	65.00
	Total	48	28.7500	17.48860	2.52426	23.6718	33.8282	.00	80.00
Better ear; Mean	Successful users	24	16.9792	7.80117	1.59241	13.6850	20.2733	2.50	30.00
	Intermittent users	10	21.7500	7.24569	2.29129	16.5667	26.9333	10.00	30.00
	Failed users	14	20.9821	8.43782	2.25510	16.1103	25.8540	7.50	30.00
	Total	48	19.1406	8.02322	1.15805	16.8109	21.4703	2.50	30.00
Worse ear; 0.5kHz	Successful users	24	53.9583	13.34974	2.72500	48.3212	59.5954	30.00	75.00
	Intermittent users	10	57.5000	11.60699	3.67045	49.1969	65.8031	30.00	70.00
	Failed users	14	51.7857	15.76353	4.21298	42.6841	60.8873	25.00	65.00
	Total	48	54.0625	13.63101	1.96747	50.1045	58.0205	25.00	75.00
Worse ear; 1kHz	Successful users	24	55.8333	10.59806	2.16332	51.3582	60.3085	35.00	80.00
	Intermittent users	10	59.0000	11.25463	3.55903	50.9489	67.0511	45.00	80.00
	Failed users	14	56.4286	14.73204	3.93730	47.9225	64.9346	20.00	85.00
	Total	48	56.6667	11.86508	1.71258	53.2214	60.1119	20.00	85.00
Worse ear; 2kHz	Successful users	24	55.6250	10.24828	2.09192	51.2975	59.9525	30.00	70.00
	Intermittent users	10	52.5000	11.36515	3.59398	44.3699	60.6301	35.00	70.00
	Failed users	14	57.8571	11.04437	2.95173	51.4803	64.2340	30.00	70.00
	Total	48	55.6250	10.65039	1.53725	52.5324	58.7176	30.00	70.00
Worse ear; 4kHz	Successful users	24	62.2917	14.36929	2.93312	56.2240	68.3593	30.00	85.00
	Intermittent users	10	52.0000	15.67021	4.95536	40.7902	63.2098	25.00	70.00
	Failed users	14	65.3571	10.82452	2.89298	59.1072	71.6070	50.00	90.00
	Total	48	61.0417	14.29024	2.06262	56.8922	65.1911	25.00	90.00
Worse ear; Mean	Successful users	24	56.9271	7.53596	1.53827	53.7449	60.1092	41.25	68.75
	Intermittent users	10	55.2500	6.99206	2.21108	50.2482	60.2518	46.25	66.25
	Failed users	14	57.8571	7.68097	2.05282	53.4223	62.2920	46.25	68.75
	Total	48	56.8490	7.37029	1.06381	54.7089	58.9891	41.25	68.75

(b) Box-plots of differences in the hearing thresholds according to two outcomes (successful-intermittent users and failed users).

		N	Mean	Standard deviation	Standard error	95% Confidence interval for mean Lower bound	95% Confidence interval for mean Upper bound	Minimum	Maximum
Better ear; 0.5kHz	Successful-Intermittent users	34	14.7059	7.58199	1.30030	12.0604	17.3514	.00	30.00
	Failed users	14	14.6429	8.19575	2.19041	9.9108	19.3749	5.00	30.00
	Total	48	14.6875	7.67742	1.10814	12.4582	16.9168	.00	30.00
Better ear; 1kHz	Successful-Intermittent users	34	14.5588	6.78318	1.16331	12.1921	16.9256	5.00	30.00
	Failed users	14	17.1429	7.52286	2.01057	12.7993	21.4864	5.00	30.00
	Total	48	15.3125	7.02626	1.01415	13.2723	17.3527	5.00	30.00
Better ear; 2kHz	Successful-Intermittent users	34	16.9118	8.07303	1.38451	14.0950	19.7286	.00	30.00
	Failed users	14	20.0000	11.09400	2.96500	13.5945	26.4055	5.00	40.00
	Total	48	17.8125	9.04517	1.30556	15.1861	20.4389	.00	40.00
Better ear; 4kHz	Successful-Intermittent users	34	27.3529	17.67704	3.03159	21.1851	33.5208	.00	80.00
	Failed users	14	32.1429	17.17716	4.59079	22.2251	42.0607	5.00	65.00
	Total	48	28.7500	17.48860	2.52426	23.6718	33.8282	.00	80.00
Better ear; Mean	Successful-Intermittent users	34	18.3824	7.84877	1.34605	15.6438	21.1209	2.50	30.00
	Failed users	14	20.9821	8.43782	2.25510	16.1103	25.8540	7.50	30.00
	Total	48	19.1406	8.02322	1.15805	16.8109	21.4703	2.50	30.00
Worse ear; 0.5kHz	Successful-Intermittent users	34	55.0000	12.79204	2.19382	50.5366	59.4634	30.00	75.00
	Failed users	14	51.7857	15.76353	4.21298	42.6841	60.8873	25.00	65.00
	Total	48	54.0625	13.63101	1.96747	50.1045	58.0205	25.00	75.00
Worse ear; 1kHz	Successful-Intermittent users	34	56.7647	10.72256	1.83890	53.0234	60.5060	35.00	80.00
	Failed users	14	56.4286	14.73204	3.93730	47.9225	64.9346	20.00	85.00
	Total	48	56.6667	11.86508	1.71258	53.2214	60.1119	20.00	85.00
Worse ear; 2kHz	Successful-Intermittent users	34	54.7059	10.51270	1.80291	51.0378	58.3739	30.00	70.00
	Failed users	14	57.8571	11.04437	2.95173	51.4803	64.2340	30.00	70.00
	Total	48	55.6250	10.65039	1.53725	52.5324	58.7176	30.00	70.00
Worse ear; 4kHz	Successful-Intermittent users	34	59.2647	15.28181	2.62081	53.9326	64.5968	25.00	85.00
	Failed users	14	65.3571	10.82452	2.89298	59.1072	71.6070	50.00	90.00
	Total	48	61.0417	14.29024	2.06262	56.8922	65.1911	25.00	90.00
Worse ear; Mean	Successful-Intermittent users	34	56.4338	7.31549	1.25460	53.8813	58.9863	41.25	68.75
	Failed users	14	57.8571	7.68097	2.05282	53.4223	62.2920	46.25	68.75
	Total	48	56.8490	7.37029	1.06381	54.7089	58.9891	41.25	68.75

(c) Comparison by the etiology of hearing loss according to three outcomes (successful users, intermittent users and failed users).

		Etiology		
		Retrocochlear/neural causes	Other unspecified causes	Total
Outcomes	Successful users	13	11	24
	Intermittent users	1	9	10
	Failed users	3	11	14
Total		17	31	48

Figure S3. Key data of box-plots of differences in the hearing thresholds according to and comparison by the etiology of hearing loss according in asymmetrical hearing loss-type 1 (AHL1) subgroup (n = 26).

(a) Box-plots of differences in the hearing thresholds according to three outcomes (successful users, intermittent users and failed users).

		N	Mean	Standard deviation	Standard error	95% Confidence interval for mean		Minimum	Maximum
						Lower bound	Upper bound		
Better ear; 0.5kHz	Successful users	11	32.7273	11.03713	3.32782	25.3124	40.1421	20.00	50.00
	Intermittent users	8	30.6250	11.78301	4.16592	20.7742	40.4758	15.00	50.00
	Failed users	7	32.8571	6.98638	2.64060	26.3958	39.3185	20.00	40.00
	Total	26	32.1154	10.01729	1.96455	28.0693	36.1615	15.00	50.00
Better ear; 1kHz	Successful users	11	34.5455	6.87552	2.07305	29.9264	39.1645	25.00	45.00
	Intermittent users	8	33.1250	10.66955	3.77226	24.2050	42.0450	15.00	50.00
	Failed users	7	38.5714	6.90066	2.60820	32.1894	44.9535	30.00	50.00
	Total	26	35.1923	8.18300	1.60482	31.8871	38.4975	15.00	50.00
Better ear; 2kHz	Successful users	11	38.6364	16.44550	4.95851	27.5881	49.6846	20.00	80.00
	Intermittent users	8	36.8750	7.03943	2.48881	30.9899	42.7601	25.00	45.00
	Failed users	7	42.8571	10.35098	3.91230	33.2841	52.4302	25.00	55.00
	Total	26	39.2308	12.38485	2.42887	34.2284	44.2331	20.00	80.00
Better ear; 4kHz	Successful users	11	49.5455	15.24050	4.59518	39.3067	59.7842	25.00	70.00
	Intermittent users	8	55.6250	13.74188	4.85849	44.1365	67.1135	30.00	75.00
	Failed users	7	54.2857	12.05148	4.55503	43.1400	65.4315	40.00	65.00
	Total	26	52.6923	13.72813	2.69231	47.1474	58.2372	25.00	75.00
Better ear; Mean	Successful users	11	38.8636	5.54680	1.67242	35.1372	42.5900	32.50	48.75
	Intermittent users	8	39.0625	6.15100	2.17471	33.9201	44.2049	32.50	50.00
	Failed users	7	42.1429	6.27969	2.37350	36.3351	47.9506	35.00	51.25
	Total	26	39.8077	5.87040	1.15128	37.4366	42.1788	32.50	51.25
Worse ear; 0.5kHz	Successful users	11	50.0000	12.24745	3.69274	41.7721	58.2279	30.00	65.00
	Intermittent users	8	61.8750	14.37694	5.08302	49.8556	73.8944	40.00	80.00
	Failed users	7	57.8571	10.35098	3.91230	48.2841	67.4302	40.00	70.00
	Total	26	55.7692	13.09139	2.56743	50.4815	61.0570	30.00	80.00
Worse ear; 1kHz	Successful users	11	56.8182	11.46140	3.45574	49.1183	64.5181	40.00	80.00
	Intermittent users	8	63.7500	7.90569	2.79508	57.1407	70.3593	55.00	75.00
	Failed users	7	60.7143	3.45033	1.30410	57.5233	63.9053	55.00	65.00
	Total	26	60.0000	9.05539	1.77591	56.3425	63.6575	40.00	80.00
Worse ear; 2kHz	Successful users	11	58.1818	17.50325	5.27743	46.4230	69.9407	25.00	80.00
	Intermittent users	8	57.5000	9.63624	3.40693	49.4439	65.5561	45.00	75.00
	Failed users	7	60.7143	7.86796	2.97381	53.4376	67.9909	45.00	70.00
	Total	26	58.6538	12.84972	2.52004	53.4637	63.8440	25.00	80.00
Worse ear; 4kHz	Successful users	11	71.8182	25.22625	7.60600	54.8710	88.7654	40.00	115.00
	Intermittent users	8	71.8750	15.33844	5.42296	59.0517	84.6983	50.00	95.00
	Failed users	7	76.4286	9.44911	3.57143	67.6896	85.1675	65.00	90.00
	Total	26	73.0769	18.60521	3.64878	65.5621	80.5917	40.00	115.00
Worse ear; Mean	Successful users	11	59.2045	6.98822	2.10703	54.5098	63.8993	47.50	67.50
	Intermittent users	8	63.7500	5.90097	2.08631	58.8167	68.6833	51.25	68.75
	Failed users	7	63.9286	3.01287	1.13876	61.1421	66.7150	58.75	68.75
	Total	26	61.8750	6.07505	1.19142	59.4212	64.3288	47.50	68.75

(b) Box-plots of differences in the hearing thresholds according to two outcomes (successful-intermittent users and failed users).

		N	Mean	Standard deviation	Standard error	95% Confidence interval for mean Lower bound	Upper bound	Minimum	Maximum
Better ear; 0.5kHz	Successful-Intermittent users	19	31.8421	11.08183	2.54235	26.5008	37.1834	15.00	50.00
	Failed users	7	32.8571	6.98638	2.64060	26.3958	39.3185	20.00	40.00
	Total	26	32.1154	10.01729	1.96455	28.0693	36.1615	15.00	50.00
Better ear; 1kHz	Successful-Intermittent users	19	33.9474	8.42927	1.93381	29.8846	38.0101	15.00	50.00
	Failed users	7	38.5714	6.90066	2.60820	32.1894	44.9535	30.00	50.00
	Total	26	35.1923	8.18300	1.60482	31.8871	38.4975	15.00	50.00
Better ear; 2kHz	Successful-Intermittent users	19	37.8947	13.05073	2.99404	31.6045	44.1850	20.00	80.00
	Failed users	7	42.8571	10.35098	3.91230	33.2841	52.4302	25.00	55.00
	Total	26	39.2308	12.38485	2.42887	34.2284	44.2331	20.00	80.00
Better ear; 4kHz	Successful-Intermittent users	19	52.1053	14.55982	3.34025	45.0877	59.1229	25.00	75.00
	Failed users	7	54.2857	12.05148	4.55503	43.1400	65.4315	40.00	65.00
	Total	26	52.6923	13.72813	2.69231	47.1474	58.2372	25.00	75.00
Better ear; Mean	Successful-Intermittent users	19	38.9474	5.64061	1.29405	36.2287	41.6661	32.50	50.00
	Failed users	7	42.1429	6.27969	2.37350	36.3351	47.9506	35.00	51.25
	Total	26	39.8077	5.87040	1.15128	37.4366	42.1788	32.50	51.25
Worse ear; 0.5kHz	Successful-Intermittent users	19	55.0000	14.14214	3.24443	48.1837	61.8163	30.00	80.00
	Failed users	7	57.8571	10.35098	3.91230	48.2841	67.4302	40.00	70.00
	Total	26	55.7692	13.09139	2.56743	50.4815	61.0570	30.00	80.00
Worse ear; 1kHz	Successful-Intermittent users	19	59.7368	10.47135	2.40229	54.6898	64.7839	40.00	80.00
	Failed users	7	60.7143	3.45033	1.30410	57.5233	63.9053	55.00	65.00
	Total	26	60.0000	9.05539	1.77591	56.3425	63.6575	40.00	80.00
Worse ear; 2kHz	Successful-Intermittent users	19	57.8947	14.36777	3.29619	50.9697	64.8198	25.00	80.00
	Failed users	7	60.7143	7.86796	2.97381	53.4376	67.9909	45.00	70.00
	Total	26	58.6538	12.84972	2.52004	53.4637	63.8440	25.00	80.00
Worse ear; 4kHz	Successful-Intermittent users	19	71.8421	21.09572	4.83969	61.6743	82.0099	40.00	115.00
	Failed users	7	76.4286	9.44911	3.57143	67.6896	85.1675	65.00	90.00
	Total	26	73.0769	18.60521	3.64878	65.5621	80.5917	40.00	115.00
Worse ear; Mean	Successful-Intermittent users	19	61.1184	6.78149	1.55578	57.8498	64.3870	47.50	68.75
	Failed users	7	63.9286	3.01287	1.13876	61.1421	66.7150	58.75	68.75
	Total	26	61.8750	6.07505	1.19142	59.4212	64.3288	47.50	68.75

(c) Comparison by the etiology of hearing loss according to three outcomes (successful users, intermittent users and failed users).

		Etiology: Retrocochlear/neural causes	Other unspecified causes	Total
Outcomes	Successful users	4	7	11
	Intermittent users	0	8	8
	Failed users	0	7	7
Total		4	22	26

(d) Comparison by the etiology of hearing loss according to three outcomes (successful users, intermittent users and failed users).

		Etiology			
		Sudden hearing loss	CNS causes	Other unspecified causes	Total
Outcomes	Successful users	4	0	7	11
	Intermittent users	0	0	8	8
	Failed users	0	0	7	7
Total		4	0	22	26

Figure S4. Key data of box-plots of differences in the hearing thresholds in asymmetrical hearing loss-type 2 (AHL2) subgroup (n = 28).

(a) Box-plots of differences in the hearing thresholds according to three outcomes for AHL2 subgroup (successful users, intermittent users and failed users).

		N	Mean	Standard deviation	Standard error	95% Confidence interval for mean		Minimum	Maximum
						Lower bound	Upper bound		
Better ear; 0.5kHz	Successful users	9	30.5556	11.57704	3.85901	21.6567	39.4545	10.00	45.00
	Intermittent users	7	30.7143	9.75900	3.68856	21.6887	39.7399	20.00	50.00
	Failed users	12	35.4167	14.53184	4.19498	26.1836	44.6498	15.00	65.00
	Total	28	32.6786	12.35899	2.33563	27.8863	37.4709	10.00	65.00
Better ear; 1kHz	Successful users	9	40.0000	8.66025	2.88675	33.3431	46.6569	25.00	55.00
	Intermittent users	7	40.0000	7.63763	2.88675	32.9364	47.0636	30.00	50.00
	Failed users	12	36.6667	10.29857	2.97294	30.1233	43.2101	20.00	55.00
	Total	28	38.5714	9.01204	1.70312	35.0769	42.0659	20.00	55.00
Better ear; 2kHz	Successful users	9	44.4444	12.61062	4.20354	34.7511	54.1378	25.00	65.00
	Intermittent users	7	42.1429	5.66947	2.14286	36.8995	47.3862	35.00	50.00
	Failed users	12	47.0833	14.05482	4.05728	38.1533	56.0133	25.00	70.00
	Total	28	45.0000	11.78511	2.22718	40.4302	49.5698	25.00	70.00
Better ear; 4kHz	Successful users	9	53.3333	15.61249	5.20416	41.3325	65.3342	25.00	75.00
	Intermittent users	7	57.1429	16.03567	6.06092	42.3123	71.9734	35.00	80.00
	Failed users	12	64.1667	12.93925	3.73524	55.9455	72.3879	45.00	90.00
	Total	28	58.9286	14.86714	2.80962	53.1637	64.6934	25.00	90.00
Better ear; Mean	Successful users	9	42.0833	7.34209	2.44736	36.4397	47.7270	32.50	52.50
	Intermittent users	7	42.5000	7.70552	2.91241	35.3736	49.6264	31.25	52.50
	Failed users	12	45.8333	10.03309	2.89630	39.4586	52.2081	33.75	65.00
	Total	28	43.7946	8.56945	1.61947	40.4718	47.1175	31.25	65.00
Worse ear; 0.5kHz	Successful users	9	66.6667	24.10913	8.03638	48.1348	85.1986	30.00	105.00
	Intermittent users	7	77.8571	10.74598	4.06160	67.9188	87.7955	65.00	95.00
	Failed users	12	79.1667	14.11533	4.07474	70.1982	88.1351	55.00	110.00
	Total	28	74.8214	17.66363	3.33811	67.9722	81.6707	30.00	110.00
Worse ear; 1kHz	Successful users	9	78.3333	18.54050	6.18017	64.0818	92.5848	50.00	105.00
	Intermittent users	7	82.1429	11.85227	4.47974	71.1813	93.1044	65.00	100.00
	Failed users	12	83.7500	11.10385	3.20540	76.6950	90.8050	70.00	105.00
	Total	28	81.6071	13.74729	2.59799	76.2765	86.9378	50.00	105.00
Worse ear; 2kHz	Successful users	9	93.8889	17.28037	5.76012	80.6060	107.1718	75.00	120.00
	Intermittent users	7	74.2857	6.72593	2.54216	68.0653	80.5062	65.00	85.00
	Failed users	12	81.2500	18.23147	5.26297	69.6663	92.8337	55.00	120.00
	Total	28	83.5714	17.15167	3.24136	76.9207	90.2222	55.00	120.00
Worse ear; 4kHz	Successful users	9	99.4444	14.45779	4.81926	88.3312	110.5577	80.00	120.00
	Intermittent users	7	83.5714	10.29332	3.89051	74.0517	93.0912	75.00	105.00
	Failed users	12	91.6667	24.71044	7.13329	75.9664	107.3669	55.00	120.00
	Total	28	92.1429	19.26562	3.64086	84.6724	99.6133	55.00	120.00
Worse ear; Mean	Successful users	9	84.5833	13.44840	4.48280	74.2460	94.9207	70.00	110.00
	Intermittent users	7	79.4643	8.09633	3.06013	71.9764	86.9521	73.75	96.25
	Failed users	12	83.9583	13.15158	3.79653	75.6022	92.3144	70.00	113.75
	Total	28	83.0357	11.96266	2.26073	78.3971	87.6743	70.00	113.75

(b) Box-plots of differences in the hearing thresholds according to two outcomes for AHL2 subgroup (successful-intermittent users and failed users).

		N	Mean	Standard deviation	Standard error	95% Confidence interval for mean		Minimum	Maximum
						Lower bound	Upper bound		
Better ear; 0.5kHz	Successful-Intermittent users	16	30.6250	10.46821	2.61705	25.0469	36.2031	10.00	50.00
	Failed users	12	35.4167	14.53184	4.19498	26.1836	44.6498	15.00	65.00
	Total	28	32.6786	12.35899	2.33563	27.8863	37.4709	10.00	65.00
Better ear; 1kHz	Successful-Intermittent users	16	40.0000	7.95822	1.98956	35.7594	44.2406	25.00	55.00
	Failed users	12	36.6667	10.29857	2.97294	30.1233	43.2101	20.00	55.00
	Total	28	38.5714	9.01204	1.70312	35.0769	42.0659	20.00	55.00
Better ear; 2kHz	Successful-Intermittent users	16	43.4375	9.95301	2.48825	38.1339	48.7411	25.00	65.00
	Failed users	12	47.0833	14.05482	4.05728	38.1533	56.0133	25.00	70.00
	Total	28	45.0000	11.78511	2.22718	40.4302	49.5698	25.00	70.00
Better ear; 4kHz	Successful-Intermittent users	16	55.0000	15.38397	3.84599	46.8025	63.1975	25.00	80.00
	Failed users	12	64.1667	12.93925	3.73524	55.9455	72.3879	45.00	90.00
	Total	28	58.9286	14.86714	2.80962	53.1637	64.6934	25.00	90.00
Better ear; Mean	Successful-Intermittent users	16	42.2656	7.24883	1.81221	38.4030	46.1283	31.25	52.50
	Failed users	12	45.8333	10.03309	2.89630	39.4586	52.2081	33.75	65.00
	Total	28	43.7946	8.56945	1.61947	40.4718	47.1175	31.25	65.00
Worse ear; 0.5kHz	Successful-Intermittent users	16	71.5625	19.72467	4.93117	61.0520	82.0730	30.00	105.00
	Failed users	12	79.1667	14.11533	4.07474	70.1982	88.1351	55.00	110.00
	Total	28	74.8214	17.66363	3.33811	67.9722	81.6707	30.00	110.00
Worse ear; 1kHz	Successful-Intermittent users	16	80.0000	15.59915	3.89979	71.6878	88.3122	50.00	105.00
	Failed users	12	83.7500	11.10385	3.20540	76.6950	90.8050	70.00	105.00
	Total	28	81.6071	13.74729	2.59799	76.2765	86.9378	50.00	105.00
Worse ear; 2kHz	Successful-Intermittent users	16	85.3125	16.68020	4.17005	76.4242	94.2008	65.00	120.00
	Failed users	12	81.2500	18.23147	5.26297	69.6663	92.8337	55.00	120.00
	Total	28	83.5714	17.15167	3.24136	76.9207	90.2222	55.00	120.00
Worse ear; 4kHz	Successful-Intermittent users	16	92.5000	14.83240	3.70810	84.5964	100.4036	75.00	120.00
	Failed users	12	91.6667	24.71044	7.13329	75.9664	107.3669	55.00	120.00
	Total	28	92.1429	19.26562	3.64086	84.6724	99.6133	55.00	120.00
Worse ear; Mean	Successful-Intermittent users	16	82.3438	11.38232	2.84558	76.2785	88.4090	70.00	110.00
	Failed users	12	83.9583	13.15158	3.79653	75.6022	92.3144	70.00	113.75
	Total	28	83.0357	11.96266	2.26073	78.3971	87.6743	70.00	113.75
